# Improving quantitative BOLD–based measures of oxygen extraction fraction using hyperoxia BOLD–derived measures of blood volume

**DOI:** 10.1002/mrm.30559

**Published:** 2025-05-20

**Authors:** Alan J. Stone, Nicholas P. Blockley

**Affiliations:** ^1^ Wellcome Center for Integrative Neuroimaging, FMRIB, Nuffield Department of Clinical Neurosciences University of Oxford Oxford UK; ^2^ Department of Medical Physics and Clinical Engineering Beaumont Hospital Dublin Ireland; ^3^ National Neuroscience Center Beaumont Hospital Dublin Ireland; ^4^ Center for Physics and Health in Medicine, School of Physics University College Dublin Dublin Ireland; ^5^ School of Life Sciences University of Nottingham Nottingham UK

**Keywords:** cerebral blood volume, hyperoxia, oxygen extraction fraction, quantitative BOLD

## Abstract

**Purpose:**

Streamlined quantitative BOLD (sqBOLD) is a refinement of the quantitative BOLD (qBOLD) technique capable of producing noninvasive and quantitative maps of oxygen extraction fraction (OEF) in a clinically feasible scan time. However, sqBOLD measurements of OEF have been reported as being systematically lower than expected in healthy brain. Because the qBOLD framework infers OEF from the ratio of the reversible transverse relaxation rate ($R_{2}^{\prime }$) and deoxygenated blood volume (DBV), this underestimation has been attributed the overestimation of DBV. Therefore, this study proposes the use of an independent measure of DBV using hyperoxia BOLD and investigates whether this results in improved estimates of OEF.

**Methods:**

Monte Carlo simulations were used to simulate the qBOLD and hyperoxia‐BOLD signals and to compare the systematic and noise‐related errors of sqBOLD and the new hyperoxia‐qBOLD (hqBOLD) technique. Experimentally, sqBOLD and hqBOLD measurements were performed and compared with TRUST (T_2_ relaxation under spin tagging)–based oximetry in the sagittal sinus.

**Results:**

Simulations showed a large improvement in the uncertainty of DBV measurements, leading to a much improved dynamic range for OEF measurements with hqBOLD. In a group of 10 healthy volunteers, hqBOLD produced measurements of OEF in cortical gray matter (OEF_hqBOLD_ = 38.1 ± 10.1%) that were not significantly different from TRUST oximetry measures (OEF_TRUST_ = 40.4 ± 7.7%), whereas sqBOLD‐derived measures (OEF_sqBOLD_ = 16.1 ± 3.1%) were found to be significantly different.

**Conclusion:**

The simulations and experiments in this study demonstrate that an independent measure of DBV provides improved estimates of OEF.

## 1 INTRODUCTION

Oxygen extraction fraction (OEF) is an important indicator of the metabolic function of brain tissue that describes the fraction of oxygen removed from arterial blood to serve oxidative metabolism. The quantitative blood oxygen–level dependent (qBOLD) technique provides the capability to noninvasively and quantitatively map OEF on a regional level.^1^, ^2^ Streamlined qBOLD (sqBOLD) is a refinement of this approach that seeks to simplify conventional qBOLD by using a prospective correction for magnetic field inhomogeneity and a fluid‐attenuated inversion recovery (FLAIR) preparation to suppress cerebral spinal fluid signal and hence reduce the number of signal compartments in the analysis.^3^

Measurements of OEF using sqBOLD are lower than expected when compared with literature values (OEF_sqBOLD_ ˜ 20%, OEF_literature_ ˜ 30%–40%).^3^ Because OEF is derived from the ratio of the irreversible transverse relaxation rate ($R_{2}^{\prime }$) and the deoxygenated blood volume (DBV), it follows that the underestimation of OEF could be due to underestimation of $R_{2}^{\prime }$ or overestimation of DBV. The sqBOLD technique uses an asymmetric spin echo (ASE) acquisition, with which $R_{2}^{\prime }$ has been measured to be in the range of 2.6–3.6 s^−1^ in gray matter (GM).^3^, ^4^, ^5^ This is consistent with previous ASE measurements^6^ of 3.5 s^−1^ and with different acquisitions such as gradient‐echo sampling of spin echo (GESSE)^2^ measuring 2.9 s^−1^. DBV has been measured by sqBOLD to be in the range of 3.6–6.7% in GM.^3^, ^4^, ^5^ This is consistent with other ASE‐based measurements^6^ of 4.3%, but not with previous measurements using GESSE^2^ of 1.8% or hyperoxia BOLD^7^ of 2.2%, suggesting that DBV overestimation might be responsible for OEF underestimation in ASE‐based qBOLD. This hypothesis is given further weight by simulations of ASE‐based qBOLD showing that diffusion, which is not incorporated in the qBOLD model, causes DBV to be overestimated.^8^ There is therefore great potential to improve the accuracy of sqBOLD by improving the accuracy of DBV measurements.

Multiparametric qBOLD introduced the concept of acquiring an independent measurement of blood volume, which is combined with a measurement of $R_{2}^{\prime }$ acquired from separate maps^9^, ^10^ of T_2_ and $T_{2}^{*}$. Here the blood volume is measured using the dynamic susceptibility contrast (DSC) technique,^11^ which requires an injection of a gadolinium‐based contrast agent. However, the DBV in the qBOLD model explicitly refers to the proportion of the blood volume that contains deoxyhemoglobin. Because DSC is sensitive to all of the vascular compartments that the contrast agent passes through and therefore is a measure of total cerebral blood volume (CBVt), for sqBOLD this DSC measure of CBV would likely drive OEF estimates even lower.

Hyperoxia BOLD provides an alternative to DSC, which is targeted specifically at measuring venous CBV (CBVv). It has been shown that the fractional change in the BOLD signal in response to the administration of oxygen is specific to CBVv and can be scaled to provide quantitative estimates using a heuristic model.^7^ The hyperoxia BOLD and qBOLD techniques rely on the same BOLD contrast. The former uses oxygen as a contrast agent by manipulating the concentration of deoxyhemoglobin in the deoxygenated blood vessels, whereas the latter relies on the additional signal attenuation due to deoxyhemoglobin at short ASE refocusing offset times. Therefore, theoretically, the CBVv from hyperoxia BOLD is equivalent to DBV from qBOLD and henceforth we will refer to both as DBV.

The aim of this study is to investigate the use of hyperoxia BOLD to improve the accuracy of OEF measurements. First, detailed simulations of the proposed technique were performed using a Monte Carlo–based approach.^8^ The potential for systematic error due to physiological variability and the effect of noise were both examined and compared with ASE‐based qBOLD. Second, experimental measurements were performed to compare the existing sqBOLD approach with the new hyperoxia‐qBOLD (hqBOLD) technique. Furthermore, comparison is made of both techniques with whole‐brain oximetry measurements from the TRUST (T_2_ relaxation under spin tagging) technique,^12^ which is an established global measure of OEF from the sagittal sinus.

## 2 METHODS

### 2.1 Simulations

Simulations of the qBOLD and hyperoxia‐BOLD signal were performed using a previously reported approach^8^ and are covered in detail in the Supporting Information. In this study, simulations of the ASE qBOLD and gradient‐echo BOLD signals were performed to match the experimental parameters described in subsequent sections. Due to the limitations of the existing Monte Carlo data, the simulated spin‐echo displacement times (τ) of the ASE measurements were required to be a multiple of 4 ms and the echo time (TE) of the BOLD measurements a multiple of 2 ms. Because the experimental values did not meet these criteria, a wider range of τ and TE values were simulated and linearly interpolated to the required values. The hyperoxia‐BOLD experiment was simulated by estimating the oxygen‐carrying capacity (CaO_2_) of arterial blood during normoxia and hyperoxia as follows:

(1) $$\mathrm{Ca}O_{2}=\phi \left[\mathrm{Hb}\right]\mathrm{Sa}O_{2}+\varepsilon \mathrm{Pa}O_{2}$$

where the constant ϕ is the oxygen‐carrying capacity of hemoglobin (1.34 mLO_2_ g^−1^); [Hb] is the hemoglobin concentration (typical value 15 gHb dl^−1^); ε is the solubility coefficient of oxygen in plasma (0.0031 mLO_2_ dl^−1^ mmHg^−1^); and the arterial oxygen saturation (SaO_2_) was estimated using the Severinghaus equation. The amount of oxygen extracted for metabolism (CmetO_2_) was assumed to be constant for normoxia and hyperoxia, enabling the venous oxygen carrying capacity (CvO_2_) to be estimated.

(2) $$\mathrm{Cv}O_{2}=\mathrm{Ca}O_{2}-\text{Cmet}O_{2}=\phi \left[\mathrm{Hb}\right]\mathrm{Sv}O_{2}+\mathrm{\varepsilon Pv}O_{2}$$

The venous partial pressure of oxygen (PvO_2_) was calculated assuming that the plasma oxygen component was nonnegligible and used to calculate the venous oxygen saturation (SvO_2_) via the Severinghaus equation. Finally, the capillary oxygen saturation (ScO_2_) was calculated as a weighted sum of the SaO_2_ and SvO_2_ (ScO_2_ = κ SaO2 + (1‐κ) SvO_2_), with a weighting factor κ = 0.4 giving a greater weighting toward venous blood.

The combined physical and physiological model of the MRI signal was used in two ways: (i) to investigate the presence of systematic error and (ii) to investigate the effect of noise on the parameter estimates. Systematic error was investigated in the absence of system noise and incorporated variations in CBVt, OEF, hematocrit (Hct), normoxic PaO_2_ (PaO_2_^norm^), and hyperoxic PaO_2_ (PaO_2_^hyper^). As in previous publications, this was achieved by randomly generating values in a predefined range (Table 1) using a uniform random number generator for all five parameters.^13^ These values were then used to generate ASE and BOLD signals using the model. This process was repeated to produce 1000 different physiological states and accompanying signals. These signals were processed to generate estimates of $R_{2}^{\prime }$ and OEF using the process described in Section 2.3. The estimation of DBV differed from the experimental method, which uses linear regression, in that single estimates of the BOLD signal at normoxia and hyperoxia were used to calculate the percentage change in the BOLD signal (δBOLD) between these two conditions.

**TABLE 1:** Simulation parameters used to define the physiological state. The standard value was used when investigating the effect of noise and the range tested reflects the ranges used to investigate systematic error.

Parameter	Standard value	Range tested	Description
CBVt	5 %	0–10 %	Total cerebral blood volume
DBV	2.3 %	0–5.7 %	Deoxygenated blood volume
OEF	40 %	0–100 %	Oxygen extraction fraction
Hct	34 %	31–43 %	Small vessel haematocrit
PaO_2_^norm^	110 mmHg	100–130 mmHg	Normoxic arterial partial pressure of oxygen
PaO_2_^hyper^	400 mmHg	370–450 mmHg	Hyperoxic arterial partial pressure of oxygen

The effect of noise was investigated by simulating signals for standard physiological values (Table 1). Gaussian random noise was then added to these signals before calculating $R_{2}^{\prime }$, DBV, and OEF. This process was repeated 20 000 times. The signal‐to‐noise ratio (SNR) for ASE and BOLD was chosen to match the distributions of DBV values observed experimentally.

For the purposes of comparison, the true value of a parameter is required. This is problematic given the definition of DBV as the vascular volume containing deoxygenated blood, as this applies to both capillaries and veins. However, the capillaries contain proportionally less deoxyhemoglobin due to a higher oxygen saturation and generate a smaller fraction of the overall qBOLD signal (i.e., the signal from capillaries is expected to increase quadratically with blood oxygenation, but the linear scaling constant is 100 times smaller than for large venous vessels).^14^ Therefore, pragmatically, DBV is defined as the blood volume within the venous vessels of the model. This is consistent with our previous simulations of the hyperoxia‐BOLD signal^7^ but not our previous simulations of the qBOLD signal.^8^ The true $R_{2}^{\prime }$ was then calculated using the following definition of DBV:

(3) $$R_{2}^{\prime }=\gamma \;\frac{4}{3}\;\pi \;\Delta \chi_{0}\;\mathrm{Hct}\;\mathrm{OEF}\;B_{0}\;\mathrm{DBV}$$

where γ is the proton gyromagnetic ratio; Δ𝝌_0_ is the susceptibility difference between oxygenated and deoxygenated red blood cells (Δ𝝌_0_ = 0.264 × 10^−6^);^15^ and B_0_ is the main magnetic field (B_0_ = 3 T) and the known Hct from the simulations. The true OEF was an input to the simulations.

### 2.2 Imaging

**FIGURE 1:**
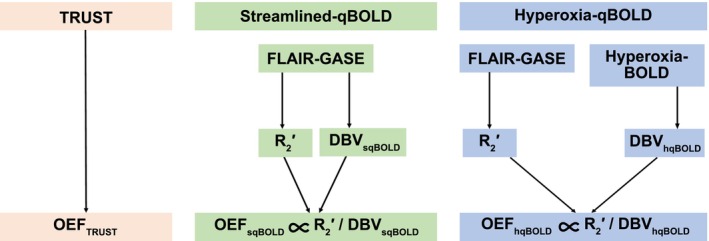
Schematic outlining the MR/physiological parameters derived from each imaging method which include the TRUST (T_2_ relaxation under spin tagging) method for measuring whole‐brain oxygen extraction fraction (OEF), the streamlined quantitative BOLD (qBOLD) technique for mapping tissue OEF and deoxygenated blood volume (DBV), and the hyperoxia‐qBOLD approach, which uses an independent measurement of DBV from hyperoxia BOLD to map OEF. BOLD, blood oxygen–level dependent.

Ten healthy participants (aged 23–41; median 29; 3 female, 7 male) were scanned with local ethics committee approval using a 3T Siemens Prisma scanner (Siemens Healthcare, Erlangen, Germany) with a 32‐channel receive‐only head coil. Figure 1 presents an overview of the imaging data acquired, the parameters derived from this imaging data, and the main comparisons performed in this study. MRI data were acquired in the following order:

TRUST oximetry measurements were made using an in‐house pulse sequence in the superior sagittal sinus (SSS) using the following parameters: field of view (FOV) = 230 mm^2^, 64 × 64 matrix, repetition time (TR)/TE = 3 s/7 ms, flip angle (FA) = 90°, GRAPPA = 3, partial Fourier = 6/8, bandwidth (BW) = 2604 Hz/px, tag gap = 25 mm, tag thickness = 100 mm, inversion time (TI) = 1050 ms. Four tag–control pairs were acquired at four different effective TEs (eTE = 0, 40, 80, and 160 ms), resulting in 32 acquisitions and a total scan duration of 1:53 min.

The sqBOLD measurements were made using a FLAIR‐GASE acquisition^16^ with the following parameters: FOV = 220 mm^2^, 96 × 96 matrix, nine 5‐mm slabs (encoded into four 1.25‐mm slices, 100% partition oversampling), 2.5‐mm gap, TR/TE = 3 s/80 ms, FA = 90°, BW = 2004 Hz/px, TI_FLAIR_ = 1210 ms, and τ = 0–66 ms in steps of 3 ms. This resulted in 23 τ‐weighted acquisitions with a total scan duration of 9:12 min.

The same ASE data were used for the hqBOLD measurements and complemented with hyperoxia‐BOLD data to independently estimate DBV. BOLD echo planar imaging data were acquired with a matched slice prescription to the FLAIR‐GASE acquisitions (FOV = 220 mm,^2^ 96 × 96 matrix, nine 5‐mm slices, 2.5‐mm gap, TR/TE = 1 s/35 ms, FA = 65°, BW = 2004 Hz/px). A prospective end‐tidal gas targeting system (RespirAct Gen 3; Thornhill Research Inc., Toronto, Canada) was used to modulate end‐tidal oxygen (petO_2_) between normoxic and hyperoxic (baseline +300 mmHg) conditions, while maintaining isocapnia (a constant carbon dioxide level). The hyperoxia paradigm lasted 10 min, during which time 600 volumes were acquired. The respiratory paradigm consisted of three 2‐min blocks of normoxia interleaved with two 2‐min blocks of hyperoxia. The combined acquisition time was 19:12 min.

A magnetization‐prepared rapid gradient‐echo data set (FOV = 174 × 192 × 192 mm, 116 × 128 × 128 matrix, TR/TI/TE = 1900/904/3.74 ms, and FA = 8°) was acquired in each subject.^17^ To aid in the registration of the FLAIR‐GASE to the structural image, an additional set of whole‐brain GASE data without a FLAIR preparation were acquired with an increased coverage in the z‐direction and a set of ASE data with τ = 0 to estimate image SNR.

### 2.3 Data analysis

The following preprocessing and analysis steps were applied to the data acquired in each subject using a combination of tools from the FMRIB Software Library (FSL)^18^ and custom scripts written using *MATLAB* (The MathWorks, Natick, MA, USA).

The tag and control images of the TRUST data were first motion‐corrected using the FMRIB linear image registration tool (MCFLIRT) (Jenkinson et al., 2002). Pairwise subtraction of the tag‐control images was performed, and the four repeats at each eTE averaged. Using the difference image at eTE = 0 ms, the four voxels with the highest signal in the SSS were identified to create a region of interest (ROI). Using the SSS ROI, the mean difference signal (ΔS) was extracted for each eTE. To calculate the T_2_ of blood (T_2b_), ΔS is plotted as a function of eTE and fitted to Eq. (4) to obtain the exponent C.^12^

(4) $$\Delta S=S_{0}e^{\mathrm{eTE}\;C}$$

The T_2_ of blood (T_2b_) was then estimated from C using Eq. (5) and assuming the T_1_ of blood (T_1b_) to be 1624 ms.

(5) $$T_{2b}=\frac{1}{\frac{1}{T_{1b}}-C}$$

Assuming a value for hematocrit in large vessels (Hct = 0.42),^19^ T_2b_ can be converted to SvO_2_ using a calibration model.^20^ Assuming arterial blood is fully saturated, an estimate of whole brain OEF is given by 1‐ SvO_2_.

For sqBOLD, the four 1.25‐mm slices of each slab of the FLAIR‐GASE data were averaged to produce nine 5‐mm slices, which were then motion‐corrected using MCFLIRT.^21^ A mask of brain tissue was created using the brain extraction tool,^22^ and further analysis was restricted to voxels within this mask. The τ‐series for each voxel were then fit using a linear system (A · x = B) to simultaneously estimate $R_{2}^{\prime }$, DBV, and a constant term representing the underlying proton density and T_2_ decay.^4^

(6) $$\left[\begin{array}{ccc} 0 & 0 & 1\\ \vdots & \vdots & \vdots \\ 1 & -\tau_{1} & 1\\ 1 & -\tau_{2} & 1\\ \vdots & \vdots & \vdots \\ 1 & -\tau_{n} & 1 \end{array}\right]\left[\begin{array}{c} \mathrm{DBV}\\ {R_{2}}^{\prime }\\ \log\left(S_{0}\right)-\mathrm{TE}\cdot R_{2} \end{array}\right]=\left[\begin{array}{c} \log\left(S\left(\tau_{0}\right)\right)\\ \vdots \\ \log\left(S\left(\tau_{1}\right)\right)\\ \log\left(S\left(\tau_{2}\right)\right)\\ \vdots \\ \log\left(S\left(\tau_{n}\right)\right) \end{array}\right]$$

Here, S(τ) is the signal intensity as a function of τ, where τ_0_ represents acquisitions at τ = 0 ms, and τ_1_ to τ_n_ are values of τ >15 ms. In Matrix A, the first row describes the short τ regime relevant to τ_0_, and subsequent rows describe the long τ regime^23^ representing τ >15 ms. Although values of τ between 3 and 12 ms are not used, this approach removes the computationally heavy process of estimating the qBOLD signal in the transition between the long and short τ regimes. The least‐squares solution was used to produce voxel‐wise estimates of $R_{2}^{\prime }$ and DBV. Parameter maps of OEF were then calculated using Δ𝝌_0_ = 0.264 × 10^−6^ ^15^ and small vessel Hct^24^ of 0.34.

(7) $$\mathrm{OEF}=\frac{R_{2}^{\prime }}{\mathrm{DBV}\;\gamma \;\frac{4}{3}\;\pi ∆\chi_{0}\;\mathrm{Hct}\;B_{0}}$$

For the multi‐parametric hqBOLD approach, a map of $R_{2}^{\prime }$ was calculated from the same data as for sqBOLD but only for images with τ >15 ms using a log‐linear least‐squares fit for $R_{2}^{\prime }$ and the constant term. The hyperoxia‐BOLD data were then analyzed in the following way. The BOLD data were motion‐corrected using MCFLIRT and then transformed into the FLAIR‐GASE image space using FLIRT.^25^ Measurements of petO_2_ were interpolated onto the 1‐s time resolution of the BOLD data and smoothed to generate a regressor of the blood oxygenation change. This model was fitted to the hyperoxia‐BOLD data on a voxel‐wise basis using the least‐squares method and used to calculate the fractional BOLD signal change to hyperoxia (*δ*BOLD). The change in petO_2_ during the hyperoxia challenge (ΔpetO_2_) was calculated by taking the mean of data within windows during baseline (Repetitions 1 to 100) and hyperoxia (Repetitions 175 to 225 and 415 to 465). These measurements were combined with a previously described heuristic model^7^ (Eq. [8]) to calculate DBV, under the assumption that ΔpetO_2_ = ΔPaO_2_ (the change in PaO_2_), A = 27 ms, B = 0.2, C = 245.1 mmHg, and D = 0.1.^7^

(8) $$\mathrm{DBV}=\left(\frac{A}{\mathrm{TE}}+B\right)\left(\frac{C}{\Delta \mathrm{Pa}O_{2}}+D\right)\;\delta \text{BOLD}$$

This heuristic model is a generalization of numerical simulation results based on observations about the relationship between the BOLD response to hyperoxia, TE, and ΔpetO_2_.

### 2.4 Regional analysis

Subject‐specific ROIs of cortical GM and white matter (WM) were created from the FLAIR‐GASE (τ = 0) data using FMRIB's automated segmentation tool (FAST)^26^ by taking advantage of the T_1_‐weighting caused by the FLAIR preparation. GM partial‐volume‐estimate maps were threshold at 50% and further refined to cortical GM by transforming the MNI structural atlas^27^ into the FLAIR‐GASE space via the magnetization‐prepared rapid gradient‐echo using FLIRT. Frontal, insula, occipital, parietal, and temporal lobes were included in the cortical mask to produce the final cortical GM ROI. WM ROIs were generated by thresholding the WM partial‐volume‐estimate maps at 100%.

### 2.5 Statistical testing

A one‐way analysis of variance was used to test the null hypothesis of no difference between the OEF estimates made using the three different techniques. Post hoc statistical testing was performed using the Tukey Kramer method (honest significant difference test) to test for differences between the estimates made using the different methods. A paired t‐test was used to test the null hypothesis of no difference between the DBV estimates made using sqBOLD and hyperoxia‐BOLD.

## 3 RESULTS

Table 2 displays ΔpetO_2_ and the change in end‐tidal partial pressure of carbon dioxide (ΔpetcO_2_) during the hyperoxia‐BOLD experiment for all participants. The mean ΔpetO_2_ was 294.7 mmHg, and the mean ΔpetcO_2_ was 0.4 mmHg.

**TABLE 2:** Baseline end‐tidal partial pressure of oxygen (petO_2_) and carbon dioxide (petcO_2_) and the changes in oxygen (ΔpetO_2_) and (ΔpetO_2_) due to the hyperoxia challenge.

Subject	petO_2_ (mmHg)	ΔpetO_2_ (mmHg)	petcO_2_ (mmHg)	ΔpetcO_2_ (mmHg)
1	122.9	321.5	34.9	1.0
2	108.9	277.8	41.7	1.2
3	109.8	295.6	43.3	0.5
4	109.2	297.0	41.3	−0.2
5	104.9	291.0	39.6	−0.1
6	109.3	268.2	39.5	2.3
7	112.0	296.9	36.3	−0.5
8	128.8	312.6	35.4	0.5
9	128.8	277.7	37.0	0.6
10	109.0	308.7	42.2	−1.1
Mean	114.4	294.7	39.1	0.4
SD	8.9	16.8	3.0	1.0

Abbreviation: SD, standard deviation.

**FIGURE 2:**
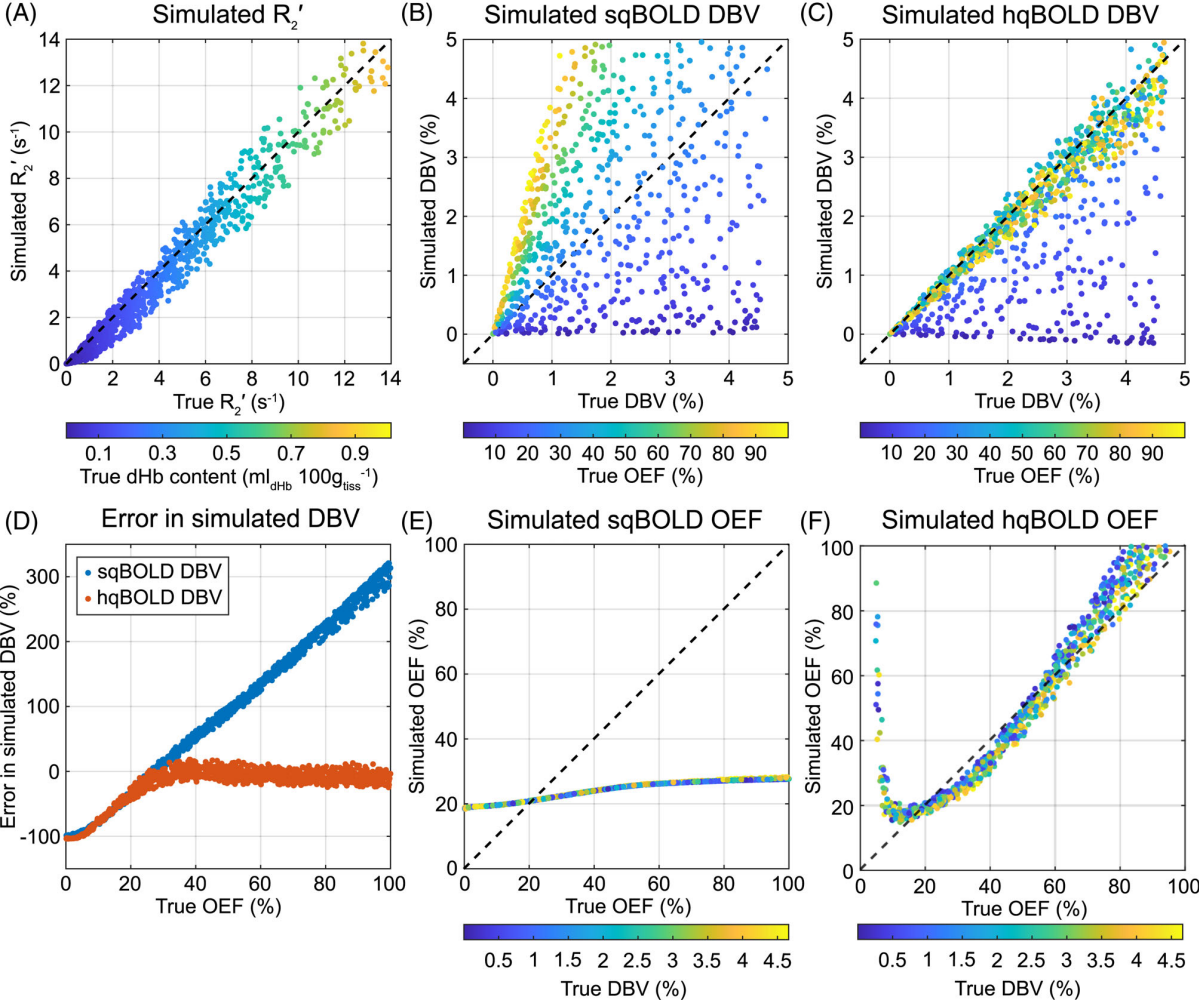
Simulations of the systematic error in the two quantitative BOLD (qBOLD) approaches. (A) Simulated $R_{2}^{\prime }$ compared with “true” $R_{2}^{\prime }$ calculated using the static dephasing qBOLD model. (B,C) Simulated streamlined qBOLD (sqBOLD) deoxygenated blood volume (DBV) and hyperoxia BOLD (hqBOLD) DBV versus true DBV, respectively. (D) Error in simulated DBV for sqBOLD and hqBOLD techniques. (E,F) Simulated OEF from sqBOLD and hqBOLD, respectively. BOLD, blood oxygen–level dependent.

Figure 2 presents the results of the simulations of the sqBOLD and hqBOLD techniques. The $R_{2}^{\prime }$ estimated from the simulated qBOLD data is plotted against the true $R_{2}^{\prime }$ based on the pragmatic definition of DBV (Figure 2A). A dashed line of unity is plotted, and the slope of the relationship between the data is estimated as 0.99 (intercept −0.24 Hz). The value of DBV_sqBOLD_ shows a large error when compared with true DBV, which increases with true OEF (Figure 2B); however, DBV_hqBOLD_ has a much lower error when OEF ≳ 30% (Figure 2C). The slope of the relationship between DBV_hqBOLD_ and true DBV is 0.78 (intercept 0.05%) for the full range of OEF values or 0.94 (intercept 0.01%) when OEF >30%. The extent of the error in DBV is shown more clearly in Figure 2D, which shows the error as a percentage of the true DBV and highlights the linear increase in DBV_sqBOLD_ error with increasing OEF and the low error for DBV_hqbold_ when OEF > 30%. The value of OEF_sqBOLD_ does increase with true OEF but has a very small dynamic range (Figure 2E). Finally, OEF_hqBOLD_ increases monotonically with true OEF for OEF > 20% with a dynamic range consistent with the full range of OEF (Figure 2F), whereas for OEF < 20%, estimates of OEF_hqBOLD_ are affected by the increasing error in DBV_hqBOLD_. It should also be noted that the uncertainty in the value of OEF is greater for hqBOLD than for sqBOLD, even considering the compressed range of the latter.

**FIGURE 3:**
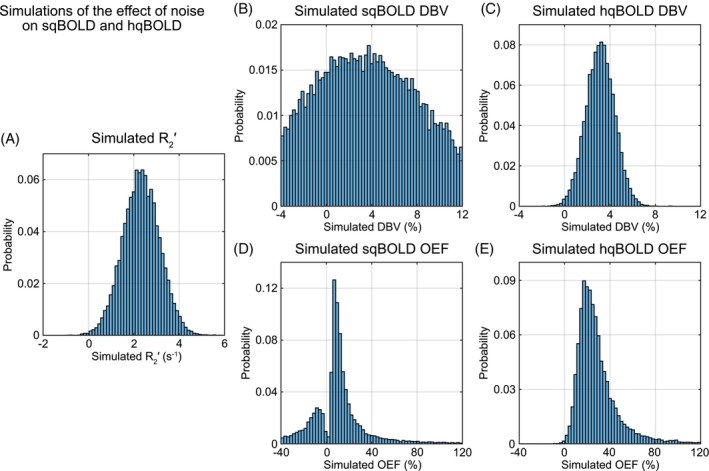
Simulations of the effect of noise for the two quantitative BOLD (qBOLD) approaches. (A) The distribution of $R_{2}^{\prime }$ values estimated from simulated qBOLD signals. (B,C) The distribution of deoxygenated blood volume (DBV) values for streamlined qBOLD (sqBOLD) and hyperoxia qBOLD (hqBOLD) techniques, respectively. (D,E) The distribution of oxygen extraction fraction (OEF) values for sqBOLD and hqBOLD, respectively. BOLD, blood oxygen–level dependent.

Figure 3 presents simulations of the effect of system noise on measurements made using sqBOLD and hqBOLD. The true $R_{2}^{\prime }$ was estimated to be 2.8 s^−1^, whereas the median value simulated was 2.3 s^−1^ (Figure 3A). The true DBV was set as 2.3% and was simulated to have a median (interquartile range) of 3.3% for sqBOLD (Figure 3B) and 3.1% for hqBOLD (Figure 3C). Finally, the true OEF was set as 40% and was estimated to be 8.7% for sqBOLD (Figure 3D) and 24.4% for hqBOLD (Figure 3E).

**FIGURE 4:**
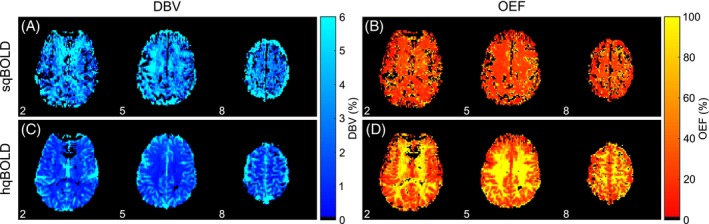
Example maps of deoxygenated blood volume (DBV) and oxygen extraction fraction (OEF) from the streamlined qBOLD (sqBOLD) technique (A,B) and the hyperoxia qBOLD (hqBOLD) method (C,D). BOLD, blood oxygen–level dependent.

Figure 4 shows parameter maps of DBV and OEF from sqBOLD and hqBOLD for three slices (Slices 2, 5, and 8) in a single subject (Subject 2). A single slice from each of the 10 participants is provided in Figure S1. The DBV_hqBOLD_ maps demonstrate an obvious contrast between GM and WM, with lower values in WM as expected. In the GM, the OEF_hqBOLD_ maps show physiologically plausible values for healthy brain tissue. However, in WM, unphysiological values of OEF are produced with a mean OEF value of 140.9%. In comparison, the DBV_sqBOLD_ and OEF_sqBOLD_ maps demonstrate little contrast between GM and WM and agree well with the initial implementation of this method.^3^

Table 3 lists the regional estimates in GM extracted from hqBOLD and sqBOLD parameter maps. These estimates are compared with values of global OEF measured using TRUST. Group mean DBV_hqBOLD_ (1.6 ± 0.5%) was found to be significantly different (*p* < 0.05) to DBV_sqBOLD_ (5.9 ± 2.1%). The one‐way analysis of variance was found to be significant (*p* < 0.001), indicating that the mean OEF values for each group are not equal. Post hoc pairwise comparisons revealed that OEF_sqBOLD_ is significantly different than OEF_hqBOLD_ (*p* < 0.001) and OEF_TRUST_ (*p* < 0.001), but that there was no significant difference between OEF_hqBOLD_ and OEF_TRUST_ (*p* = 0.77).

**TABLE 3:** Parameter estimates of deoxygenated blood volume (DBV) and oxygen extraction fraction (OEF) calculated using streamlined quantitative BOLD (sqBOLD), hyperoxia qBOLD (hqBOLD), and T_2_ relaxation under spin tagging (TRUST).

	sqBOLD			hqBOLD			TRUST
Subject	$R_{2}^{\prime }$ (s^−1^)	DBV (%)	OEF (%)	R_2_′ (s^−1^)	DBV (%)	OEF (%)	OEF (%)
1	2.1 (1.9)	2.9 (5.0)	15.6 (25.9)	2.1 (1.9)	1.2 (1.9)	40.7 (88.1)	38.6
2	2.1 (1.5)	1.9 (4.4)	15.1 (38.2)	2.1 (1.5)	2.5 (1.9)	25.0 (26.1)	34.5
3	2.8 (2.3)	3.8 (8.7)	12.6 (22.5)	2.8 (2.3)	1.4 (1.6)	50.5 (77.3)	34.2
4	3.1 (1.7)	4.0 (3.5)	22.5 (17.5)	3.1 (1.7)	1.5 (1.5)	55.7 (60.6)	41.5
5	1.9 (2.0)	3.0 (5.4)	13.5 (21.9)	1.9 (2.0)	1.5 (1.5)	34.3 (50.7)	27.4
6	2.6 (1.8)	4.3 (4.7)	16.5 (18.1)	2.6 (1.8)	2.6 (2.0)	30.2 (31.2)	36.5
7	2.7 (1.9)	4.0 (4.8)	17.2 (18.9)	2.7 (1.9)	1.2 (1.2)	63.4 (79.3)	53.7
8	3.1 (2.4)	4.5 (5.0)	19.7 (20.9)	3.1 (2.4)	1.5 (1.7)	53.1 (76.7)	44.7
9	2.6 (2.9)	3.5 (7.4)	13.5 (26.0)	2.6 (2.9)	1.1 (1.7)	45.2 (98.6)	49.0
10	4.1 (3.8)	6.9 (10.3)	15.0 (12.7)	4.1 (3.8)	1.6 (2.0)	58.6 (111.7)	45.6
Mean	2.7	5.9	16.1	2.2	1.6	45.7	40.6
SD	0.6	2.1	3.1	0.7	0.5	12.8	7.9

*Note*: Median values in global gray matter are shown for each subject and presented alongside the group mean and standard deviation (SD).

Abbreviations: BOLD, blood oxygen–level dependent; SD, standard deviation.

**FIGURE 5:**
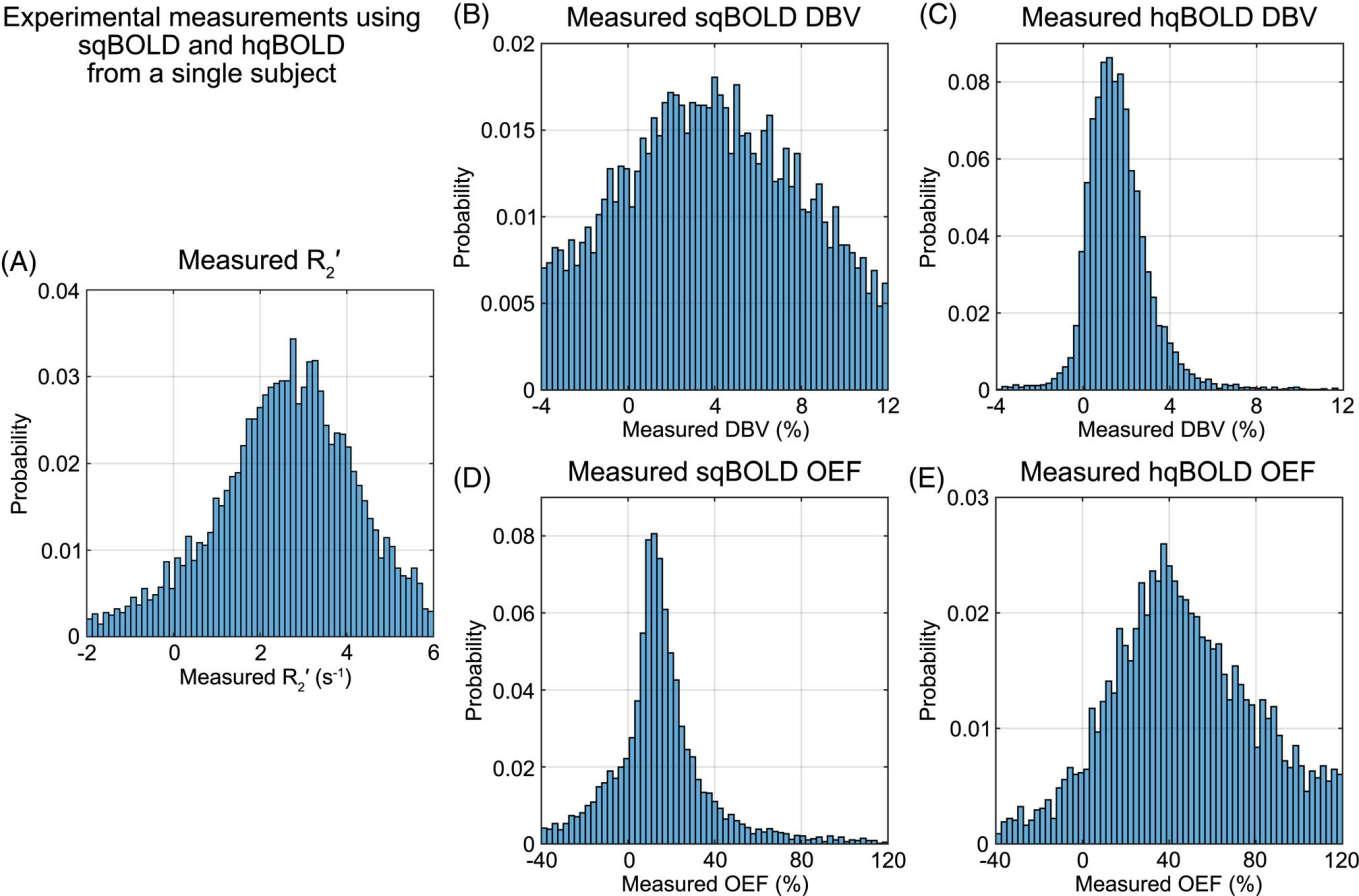
Histograms of experimental quantitative (qBOLD) measurements in gray matter. (A) The distribution of $R_{2}^{\prime }$ values. (B,C) The distribution of deoxygenated blood volume (DBV) values for streamlined qBOLD (sqBOLD) and hyperoxia qBOLD (hqBOLD) techniques, respectively. (D,E) The distribution of oxygen extraction fraction (OEF) values for sqBOLD and hqBOLD, respectively.

Figure 5 shows histograms of DBV_hqBOLD_, OEF_hqBOLD_, DBV_sqBOLD_, and OEF_sqBOLD_ voxel values within GM for one example participant (Subject 3). These histograms show the distribution of voxel values that underlie the measurements presented in Table 3. The value DBV_hqBOLD_ exhibits a narrower distribution of voxel values compared with DBV_sqBOLD_, and OEF_hqBOLD_ displays a higher median OEF (Table 3) and a broader distribution of values compared with OEF_sqBOLD_. Histograms for all 10 participants are presented in Figure S2.

**FIGURE 6:**
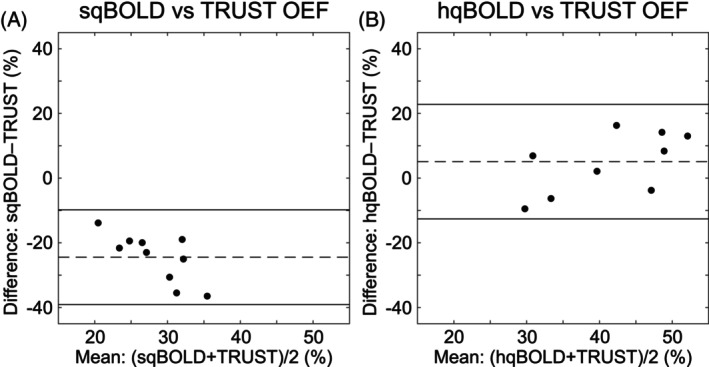
Bland–Altman plot comparing hyperoxia quantitative BOLD (hqBOLD) measures of oxygen extraction fraction (OEF) in global gray matter with streamlined qBOLD (sqBOLD) and TRUST (T_2_ relaxation under spin tagging) measures of OEF.

Figure 6 shows OEF_TRUST_ compared with OEF_hqBOLD_ and OEF_sqBOLD_. Bland–Altman plots demonstrate a large bias between OEF_TRUST_ and OEF_sqBOLD_ of −24.5% points, whereas OEF_TRUST_ and OEF_hqBOLD_ are in better agreement with a smaller bias of 5.1 points between techniques.

To understand the unphysiological OEF in WM, Table 4 compares the ratio of the GM to WM values of $R_{2}^{\prime }$, DBV_sqBOLD_, and DBV_hqBOLD_. GM $R_{2}^{\prime }$ was found to be 22% lower than WM; GM DBV_sqBOLD_ was found to be 29% lower than WM; and GM DBV_hqBOLD_ was found to be 234% higher than WM.

**TABLE 4:** To investigate the unphysiological values of oxygen extraction fraction (OEF) found in white matter (WM), the following ratios were calculated with respect to gray matter (GM).

Subject	R_2_′_GM_/ R_2_′_WM_	DBV_sqBOLD‐GM_/ DBV_sqBOLD‐WM_	DBV_hqBOLD‐GM_/ DBV_hqBOLD‐WM_
1	0.64	0.60	2.76
2	0.77	0.48	3.01
3	0.80	0.74	3.07
4	0.76	0.70	2.63
5	0.71	0.74	2.99
6	0.84	0.89	2.82
7	0.86	0.80	3.61
8	0.76	0.68	3.92
9	0.90	0.74	3.04
10	0.74	0.72	4.10
Mean	0.78	0.71	3.19
Standard deviation	0.08	0.11	0.51

*Note*: First, the ratio of the reversible transverse relaxation rate, R_2_′, in the GM (R_2_′_GM_) to the WM (R_2_′_WM_). Second, the ratio of the DBV from sqBOLD in the GM (DBV_sqBOLD‐GM_) to the WM (DBV_sqBOLD‐WM_). Finally, the ratio of the DBV from hqBOLD in the GM (DBV_hqBOLD‐GM_) to the WM (DBV_hqBOLD‐WM_).

Abbreviations: BOLD, blood oxygen–level dependent; DBV, deoxygenated blood volume; hqBOLD, hyperoxia quantitative BOLD; sqBOLD, streamlined quantitative BOLD.

## 4 DISCUSSION

The aim of this study was to investigate whether an independent measure of DBV based on the hyperoxia‐BOLD signal could improve the accuracy of qBOLD measurements of OEF. Simulations of this new technique, referred to here as hqBOLD, predicted a considerable improvement in estimates of both DBV and OEF compared with the existing sqBOLD technique. Experimentally, measurements of OEF acquired using hqBOLD and sqBOLD were compared with whole‐brain oximetry measurements from TRUST. The hqBOLD measurements of OEF were found to be in the range 23% to 53%, in good agreement with literature values of OEF in healthy cortical GM (˜35%–55%).^28^ Although sqBOLD measurements of OEF were found to be systematically low (range 12%–22%) (Table 3), no significant difference was found between GM hqBOLD OEF measurements and TRUST OEF measurements (range 28%–53%).

### 4.1 Simulations

An accelerated Monte Carlo framework that scales and combines results from single‐vessel‐radius simulations was used to examine systematic and noise related errors. Simulations of the sqBOLD technique were consistent with previous results,^8^ namely that $R_{2}^{\prime }$ is linearly related to the true $R_{2}^{\prime }$ estimated from Eq. (3), that DBV_sqBOLD_ is a function of the OEF, and this leads to a reduced dynamic range of OEF_sqBOLD_. One difference between this previous study and the current work is the definition of true DBV. As noted previously, no strict definition of DBV exists, because the level of deoxygenation varies across the vasculature and the way that deoxygenation is translated into the BOLD signal are affected by the vessel radius. Hence, DBV can be pragmatically described as either all vessels containing deoxyhemoglobin (capillaries + veins) or only venous vessels. The effect of moving from the former definition in the previous study to the latter in the current study is a greater correspondence between the simulated measurement of $R_{2}^{\prime }$ with the true $R_{2}^{\prime }$ (slope 0.99). This would suggest that the deoxygenated blood in the capillaries only makes a small contribution to the measured $R_{2}^{\prime }$.

Simulations of DBV_hqBOLD_ provide an independent confirmation of the heuristic model (Eq. [8]) for scaling the hyperoxia BOLD signal change to DBV. This heuristic was developed using an entirely different model to the one presented here based on analytical models of three vascular compartments.^7^ In this previous work, a narrower range of healthy OEF values was simulated (35%–55%). Despite this, Eq.(8) generalizes well to the much larger OEF range simulated in this study (0%–100%), with minimal error in DBV_hqBOLD_ when OEF is greater than approximately 30% (Figure 2D). This is emphasized by the close correspondence between DBV_hqBOLD_ and true DBV with a slope of 0.95 for OEF > 30% (Figure 2C). Therefore, when combined with the simulated measurement of $R_{2}^{\prime }$ and Eq. (7), the dynamic range of OEF_hqBOLD_ is markedly improved.

Using noise levels chosen to match the experimentally observed distribution of DBV, the simulations show that for a single physiological state, the median DBV is similar for both techniques and overestimates the true value by about 40% (Figure 3B,C). However, this does not entirely explain why the OEF values are found to be lower than the true OEF in the no‐noise simulations of Figure 2, particularly for sqBOLD. This underestimation can be explained by the fact that the DBV distribution must be inverted in Eq. (7) to calculate OEF. When the variance in the DBV distribution is small, then the median of the inverted distribution approaches the inverse of the distribution's median. As the variance increases, the median of the inverted DBV distribution approaches 0, resulting in the underestimation of OEF. Hence, to improve the accuracy of OEF, an estimate of DBV with both accuracy (low systematic error) and precision (low variance) is required. This effect is explained graphically in Figure S3.

### 4.2 Gray matter

Cortical GM measurements of DBV_hqBOLD_ (1.8 ± 0.6%) were significantly different (*p* < 0.05) than DBV_sqBOLD_‐derived measurements (3.9 ± 1.3%), with DBV_sqBOLD_ 2.2 times higher than DBV_hqBOLD_ on average. The sqBOLD measurements are consistent with previous implementations^3^ (3.6 ± 0.4%), whereas the hqBOLD estimates are slightly lower than past measurements^7^ (2.2 ± 0.4%). The hyperoxia‐BOLD implementation in the current study differs in two ways from previous work: A shorter TR was used (down from 3 s to 1 s), and the hyperoxic gas mixture was presented differently. Automated gas delivery was used in this study, which benefits from isocapnic control and faster transitions between oxygen levels, compared with a two‐tube nasal cannula used in the previous study. An uncontrolled hyperoxic challenge, as in the latter case, can result in hyperventilation and associated hypocapnia.^29^ However, hypocapnia should reduce the amplitude of the BOLD response to hyperoxia; hence, we would expect the previous measurements to be lower than the current. Alternatively, a short TR could result in a decrease in the BOLD response to hyperoxia due to decrease in arterial blood T_1_ due to the presence of paramagnetic oxygen dissolved in arterial blood plasma. However, detailed study of this issue has predicted that T_1_ effects are negligible when compared with the changes in deoxyhemoglobin elicited by hyperoxia.^30^

### 4.3 Validation

The TRUST technique provides a way to measure whole‐brain OEF with few assumptions providing a way to benchmark sqBOLD and hqBOLD. Bland–Altman plots demonstrate that measurements of GM OEF made with hqBOLD agree well with TRUST oximetry across the group (Figure 6B). The bias between the two methods was measured as −2.4%. In contrast, sqBOLD measures of OEF in GM demonstrate poor agreement and a large bias of −24.3% when compared with TRUST oximetry (Figure 6A).

### 4.4 White matter

The ratio of GM to WM DBV was measured as 0.7 for sqBOLD and 3.3 for hqBOLD. There are few studies in the literature that measure DBV quantitatively. A GM‐to‐WM DBV ratio of 2.29 was measured using qBOLD with the GESSE pulse sequence.^2^ Alternatively, a study of CBVt measured a GM‐to‐WM CBVt ratio of 2.38. Both values are of a similar order to that measured using hyperoxia BOLD in the current study. In contrast, DBV measured using sqBOLD in WM appears unphysiological, given that it would suggest that WM has a higher vascular density than GM.

Under the assumptions that (i) deoxyhemoglobin is the dominant source of magnetic susceptibility in the voxel and that (ii) the blood vessels are uniformly and randomly distributed, Eq. (3) describes $R_{2}^{\prime }$ as a function of OEF and DBV. Furthermore, OEF is generally found to be the same in GM and WM in positron emission tomography (PET) measurements.^31^ Therefore, under these conditions, the ratio of GM to WM $R_{2}^{\prime }$ would be expected to scale in the same way as DBV. Hence, we would expect the $R_{2}^{\prime }$ of GM to be 2.29–3.31 times higher than for WM, but the ratio was measured to be 0.78. Note that similarly small but different ratios have been found with other $R_{2}^{\prime }$ mapping sequences, which may reflect differential sensitivity to myelin/deoxyhaemoglobin.^32^ A recent investigation of $R_{2}^{\prime }$ in WM has found that these two assumptions are likely to be violated.^33^ This study followed a multiparametric qBOLD approach whereby $R_{2}^{\prime }$ is estimated from maps of R_2_ and R_2_* and estimated the angle between the primary eigenvector of WM voxels and B_0_ using DTI. The value of $R_{2}^{\prime }$ was found to increase as the angle increased. This orientation effect was considered by the authors to be a function of the preferential alignment of blood vessels parallel to WM fiber tracks and the magnetic susceptibility effects arising from the myelin‐rich WM fibers. Although orientation undoubtedly has a role at the voxel level, in the measurements presented in the current study, this is likely to have been averaged out over the WM ROI. However, the additional magnetic susceptibility effect from myelin will remain and contribute to the measured $R_{2}^{\prime }$. Note that this does not affect the hyperoxia‐BOLD DBV measurements, as this technique relies on the change in BOLD signal in response to a hyperoxic challenge and was found to measure a ratio of GM to WM consistent with CBVt measurements.

### 4.5 Limitations and future work

The simulations of the effect of noise were only performed for a single value of OEF and DBV. Although several strategies were used to accelerate the simulations approach, performing the noise simulations for multiple values of OEF and DBV would be computationally demanding and hence not possible in this study.

It is also worth noting some of the relevant limitations of the protocols used in this study. First, the discrepancy in total acquisition time between sqBOLD (9:12 min) and hqBOLD (29:12 min) may at first glance appear to be an unfair comparison from a SNR perspective. However, as the simulations show, the underestimation of OEF is not due to a limitation in SNR but is caused by a systematic error. This study focused on reducing this systematic error, and future efforts will be directed toward reducing the overall scan duration. Some of this additional scan time is a result of the hyperoxia challenge, which also increases the complexity of the technique and may hinder clinical uptake. However, inclusion of an accurate DBV estimate is critical for the accurate estimation of OEF.

The sqBOLD protocol used here has only minor differences compared with previous studies (i.e., a slightly higher density of sampling at long τ values). We have shown that the long TE used here (TE = 80 ms) results in increased signal loss at the spin echo, due to the scale of the vessel distribution approaching the diffusion narrowing regime, causing DBV to be overestimated.^8^ Unfortunately, acquisition of the data in this study commenced before this simulation study had been completed; hence, any improvements could not be implemented. Similarly, we have developed a Bayesian analysis tool for sqBOLD data, which has been shown to reduce the variance in parameter estimates.^5^ However, because a tool to analyze the hqBOLD data in a Bayesian framework was not available, we chose to use least‐squares fitting for both techniques. We remain hopeful that an optimized sqBOLD protocol coupled with Bayesian model fitting can overcome some of the inaccuracy of the protocol used here.

The TRUST protocol used in this study had a longer TE (7 ms) than recommended in a recent study^34^ (3.6 ms). This has been shown to result in an overestimation of T_2b_ that leads to a slight overestimation in OEF of approximately 3%–4%. However, this overestimation was shown to be dependent on image SNR; hence, the number of averages acquired in this study was increased from the recommended number of three to four.

## 5 CONCLUSION

It has been shown by simulation and experiment that introducing an independent measure of DBV into the qBOLD framework provides improved estimates of OEF. Although multiparametric qBOLD has previously used an independent measure of CBVt from DSC, this is not the relevant blood volume in the context of qBOLD. Hyperoxia BOLD provides a quantitative and specific measure of DBV, such that the quantification of OEF is also improved. With further refinement, this method has the potential to provide a clinically relevant measure of tissue oxygenation.

## Supporting information


**Figure S1.** A single slice of the streamlined quantitative BOLD (sqBOLD) and hyperoxia quantitative BOLD (hqBOLD) data of each of the participants. (A,B) Deoxygenated blood volume (DBV). (C,D) Oxygen extraction fraction (OEF) maps. BOLD, blood oxygen–level dependent.
**Figure S2.** Histograms of experimental quantitative BOLD (qBOLD) measurements in gray matter for all subjects. (A) The distribution of $R_{2}^{\prime }$ values. (B,C) The distribution of deoxygenated blood volume (DBV) and oxygen extraction fraction (OEF) values for hyperoxia quantitative BOLD (hqBOLD). (D,E) The distribution of DBV and OEF values for streamlined‐qBOLD (sqBOLD). BOLD, blood oxygen–level dependent.
**Figure S3.** Demonstration of the effect of increasing variance in the measurement of deoxygenated blood volume (DBV) on the estimate of oxygen extraction fraction (OEF). This figure was created by assuming OEF = 40% and DBV = 2%. Gaussian random noise with standard deviation σ was added to the DBV value as a fraction of the mean value μ of DBV. When the noise level is relatively small compared with the mean value of DBV (C), the estimated OEF values are clustered around the simulated OEF of 40%. As the noise level increases (B), the distribution of OEF values becomes broader and skewed, and the median value shifts to a lower value than the true value. When the noise level is very high (A), the median value is highly shifted toward zero. Hence, the higher noise level present in the sqBOLD estimate of DBV causes a greater level of underestimation than the lower‐noise hqBOLD measurements.

## Data Availability

The code used to generate the simulation results can be downloaded from the Zenodo repository, doi: https://doi.org/10.5281/zenodo.15089305. The raw imaging data can be accessed via the Oxford Research Archive repository, doi: http://doi.org/10.5287/bodleian:X59eGb0pv. The code used to analyze these can be accessed via the Zenodo archive, doi: https://doi.org/10.5281/zenodo.14814607.
