# Supplementary material for: Improving quantitative BOLD–based measures of oxygen extraction fraction using hyperoxia BOLD–derived measures of blood volume
Source: Magn Reson Med. 2025 May 20;94(4):1700–13. doi: 10.1002/mrm.30559 (PMC12309898; doi:10.1002/mrm.30559)
Supplement: Supplementary file 1 — Figure S1. A single slice of the streamlined quantitative BOLD (sqBOLD) and hyperoxia quantitative BOLD (hqBOLD) data of each of the participants. (A,B) Deoxygenated blood volume (DBV). (C,D) Oxygen extraction fraction (OEF) maps. BOLD, blood oxygen–level dependent. Figure S2. Histograms of experimental quantitative BOLD (qBOLD) measurements in gray matter for all subjects. (A) The distribution of R2′ values. (B,C) The distribution of deoxygenated blood volume (DBV) and oxygen extraction fraction (OEF) values for hyperoxia quantitative BOLD (hqBOLD). (D,E) The distribution of DBV and OEF values for streamlined‐qBOLD (sqBOLD). BOLD, blood oxygen–level dependent. Figure S3. Demonstration of the effect of increasing variance in the measurement of deoxygenated blood volume (DBV) on the estimate of oxygen extraction fraction (OEF). This figure was created by assuming OEF = 40% and DBV = 2%. Gaussian random noise with standard deviation σ was added to the DBV value as a fraction of the mean value μ of DBV. When the noise level is relatively small compared with the mean value of DBV (C), the estimated OEF values are clustered around the simulated OEF of 40%. As the noise level increases (B), the distribution of OEF values becomes broader and skewed, and the median value shifts to a lower value than the true value. When the noise level is very high (A), the median value is highly shifted toward zero. Hence, the higher noise level present in the sqBOLD estimate of DBV causes a greater level of underestimation than the lower‐noise hqBOLD measurements. [file MRM-94-1700-s001.pdf]

Simulation Methodology

In the following two sections, the theory and simulations are briefly described. A more complete explanation can be found in a previous report[1].

Theory

The qBOLD signal was simulated following a Monte Carlo approach by repeating the following three steps for each simulated proton.

Step 1: Generate a system of vessels. The origin points (*O*) of the vessels were randomly selected such that half were placed on the surface of a spherical universe and half were placed within the sphere, following previous work[2]. A uniform distribution of points on the surface was ensured by using a normally distributed random number generator and scaling by the radius of the universe (R_s), whilst uniform density within the sphere was ensured by the scaling factor *U* which was selected from a uniform random number generator.

$$(O_1, O_2, O_3) = \begin{cases} R_s \dfrac{(X_1, X_2, X_3)}{\sqrt{X_1^2 + X_2^2 + X_3^2}}, & \text{on sphere surface} \\ R_s\, U^{1/3} \dfrac{(X_1, X_2, X_3)}{\sqrt{X_1^2 + X_2^2 + X_3^2}}, & \text{within sphere} \end{cases} \quad \text{(S1)}$$

Vessels with a single radius, R_c, were placed at the vessel origin points described by Eq. (S1) and modelled as randomly oriented infinitely long cylinders. Vessels were added to the system until the target blood volume fraction (V_f) was reached.

Step 2: Proton random walk. Each proton was initially placed at the spherical universe. The proton was then allowed to take random steps at a time interval of Δt with mean 0 and standard deviation σ with diffusion coefficient, D.

$$\sigma = \sqrt{2\, D\, \Delta t} \quad \text{(S2)}$$

Step 3: Estimate the phase accrued at each step. During each time interval the proton accrued phase, $\Delta\phi$, which was calculated by summing up the magnetic field generated by all *N* vessels[3],

$$\Delta\phi = 2\pi\, \gamma\, B_0\, \Delta t\, (1 - Y)\, Hct\, \Delta\chi \sum_{i=1}^{N} \left(\frac{R_c}{r_i}\right)^2 \cos 2\varphi_i \sin^2 \theta_i\ , \qquad r_i \geq R_c \quad \text{(S3)}$$

where θ is the angle of the vessel with respect to B_0, φ is the angle with respect to the projection of B_0 onto a plane orthogonal to the vessel, r_i is the perpendicular distance to the vessel and *Y* is the blood oxygen saturation. Only extravascular signal was simulated. By combining the phase accrued in each interval it is possible to simulate the phase evolution ϕ as a function of τ for the ASE and GRE pulse sequences. In the case of the former, the phase from each interval was added up until $(TE - \tau)\ /\ 2$ and then subtracted for intervals up to TE, whilst for the latter the phase was merely summed up to TE. The phase evolution of *P* protons was then summed to simulate the decay of the extravascular ASE or GRE signal[4],

$$S_{EV}(TE, \tau) = \left| \frac{1}{P} \sum_{k=1}^{P} e^{i\, \phi(\tau)} \right| e^{-\frac{TE}{T_{2,t}}} \quad \text{(S4)}$$

where $T_{2,t}$ is the underlying tissue T_2. The intravascular signal, S_{IV}, was modelled using an analytical model of the blood signal[5] described by,

$$S_{IV}(TE, \tau) = \exp\left\{-\frac{\gamma^2}{2} G_0 \tau_D^2 \left[\frac{TE}{\tau_D} + \left(\frac{1}{4} + \frac{TE}{\tau_D}\right)^{\frac{1}{2}} + \frac{3}{2} - 2\left(\frac{1}{4} + \frac{TE-(TE-\tau)/2}{\tau_D}\right)^{\frac{1}{2}} - 2\left(\frac{1}{4} + \frac{(TE-\tau)/2}{\tau_D}\right)^{\frac{1}{2}}\right]\right\} \exp\left(-\frac{TE}{T_{2,b|0}}\right). \quad \text{(S5)}$$

Here $\tau_D = R_{rbc}^2/D_b$, where R_{rbc} is the characteristic size of red blood cells and D_b is the diffusion coefficient of blood, $T_{2,b|0}$ is the intrinsic T_2 of blood (measured when the blood is fully oxygenated) and G_0 is the mean square field inhomogeneity in blood[6],

$$G_0 = \frac{4}{45} Hct\,(1 - Hct)(4\,\pi\,\Delta\chi\,(0.95 - Y)\,B_0)^2, \quad \text{(S6)}$$

where the value of 0.95 represents the red blood cell oxygen saturation which is equal to the susceptibility of plasma[7]. The total signal, S_{TOT}, was then calculated as a volume weighted sum of the intra- and extravascular signals.

$$S_{TOT} = (1 - V_f)\,S_{EV} + V_f\,S_{IV} \quad \text{(S7)}$$

Methods

The tissue signal was simulated using the theory above. The extravascular signal decay was simulated using Monte Carlo simulations incorporating the effect of diffusion at 3 T with a diffusion coefficient, D, of 1 $\mu m^2 ms^{-1}$. The radius of the spherical universe was set to maintain a similar number of blood for different vessel radii (N~1,300). Protons were allowed to move with a step size, Δt, of 20 µs which was downsampled to 200 µs. The perpendicular distances of each of the vessels (r_i) to the proton were calculated and if the proton was considered to move close to a vessel, defined as when $R_c^2/r_i^2 > 0.04$, the perpendicular distance was recalculated using the original 20 µs time step to better sample the rapid magnetic field variation expected close to vessels. Protons that were found to pass inside a vessel were discarded in order to simulate non-permeable vessels. The phase was saved in 2 ms intervals and allowed to evolve up to 120 ms. A new system of vessels was generated for each proton and a total of 10,000 protons were simulated. However, only the first 5,000 protons that did not pass inside a vessel were used to calculate S_{EV} using Eq. (S4) with $T_{2,t}$=80 ms. Intravascular signal was simulated using Eqs. (S5) and (S6) with the following parameters[6]: $T_{2,b|0}$=189 ms, R_{rbc}=2.6 µm and D_b=2 $\mu m^2 ms^{-1}$. The total signal was then calculated using Eq. (S7).

Due to the computational intensity of the Monte Carlo approach, several approaches were used to accelerate these simulations. Firstly, we have previously shown that different oxygenation levels can be simulated by scaling existing Monte Carlo simulation results[8]. This is possible because the phase is a linear function of blood oxygenation, enabling the phase evolution of the ensemble to be scaled by dividing out the nominal oxygenation and multiplying in the target oxygenation. Secondly, different volume fractions can be simulated from SEV calculated using Eq. (S4). For a given vessel radius a shape function, $f(R_c,\tau)$, for the simulated V_f can be calculated using Eq. (S8) and then used to scale for a target volume fraction[9,10].

$$S_{EV}(R_c,\tau) = \exp\left[-V_f(R_c) f(R_c,\tau)\right] \quad \text{(S8)}$$

Thirdly, a system with multiple vessel radii can be simulated by combining multiple single vessel radius simulations of the extravascular signal[9,10]. The combined extravacular signal, S_{EV}^{MULTI}, is the product of the signals from the single vessel signals.

$$S_{EV}^{MULTI} = \prod_{k=1}^{M} S_{EV}(k) \quad \text{(S9)}$$

References

1. Stone AJ, Holland NC, Berman AJL, Blockley NP. Simulations of the effect of diffusion on asymmetric spin echo based quantitative BOLD: An investigation of the origin of deoxygenated blood volume overestimation. *NeuroImage*. 2019;201(March):116035. doi:10.1016/j.neuroimage.2019.116035

2. Dickson JD, Ash TWJ, Williams GB, et al. Quantitative BOLD: the effect of diffusion. *Journal of Magnetic Resonance Imaging*. 2010;32(4):953-961.

3. Boxerman JL, Hamberg LM, Rosen BR, Weisskoff RM. MR contrast due to intravascular magnetic susceptibility perturbations. *Magnetic Resonance in Medicine*. 1995;34(4):555-566.

4. Weisskoff RM, Zuo CS, Boxerman JL, Rosen BR. Microscopic susceptibility variation and transverse relaxation: theory and experiment. *Magnetic Resonance in Medicine*. 1994;31(6):601-610.

5. Berman AJL, Pike GB. Transverse signal decay under the weak field approximation: Theory and validation. *Magnetic Resonance in Medicine*. 2018;80(1):341-350. doi:10.1002/mrm.27035

6. Berman AJL, Mazerolle EL, MacDonald ME, Blockley NP, Luh WM, Pike GB. Gas-free calibrated fMRI with a correction for vessel-size sensitivity. *Neuroimage*. 2018;169:176-188.

7. Spees WM, Yablonskiy DA, Oswood MC, Ackerman JJ. Water proton MR properties of human blood at 1.5 Tesla: magnetic susceptibility, T1, T2, T2*, and non-Lorentzian signal behavior. *Magnetic Resonance in Medicine*. 2001;45(4):533-542.

8. Blockley NP, Jiang L, Gardener AG, Ludman CN, Francis ST, Gowland PA. Field strength dependence of R 1 and R2* relaxivities of human whole blood to prohance, vasovist, and deoxyhemoglobin. *Magnetic Resonance in Medicine*. 2008;60(6):1313-1320. doi:10.1002/mrm.21792

9. Dickson JD, Ash TWJ, Williams GB, Sukstanskii AL, Ansorge RE, Yablonskiy DA. Quantitative phenomenological model of the BOLD contrast mechanism. *Journal of magnetic resonance (San Diego, Calif : 1997)*. 2011;212(1):17-25.

10. Kiselev VG, Posse S. Analytical model of susceptibility-induced MR signal dephasing: effect of diffusion in a microvascular network. *Magnetic Resonance in Medicine*. 1999;41(3):499-509.

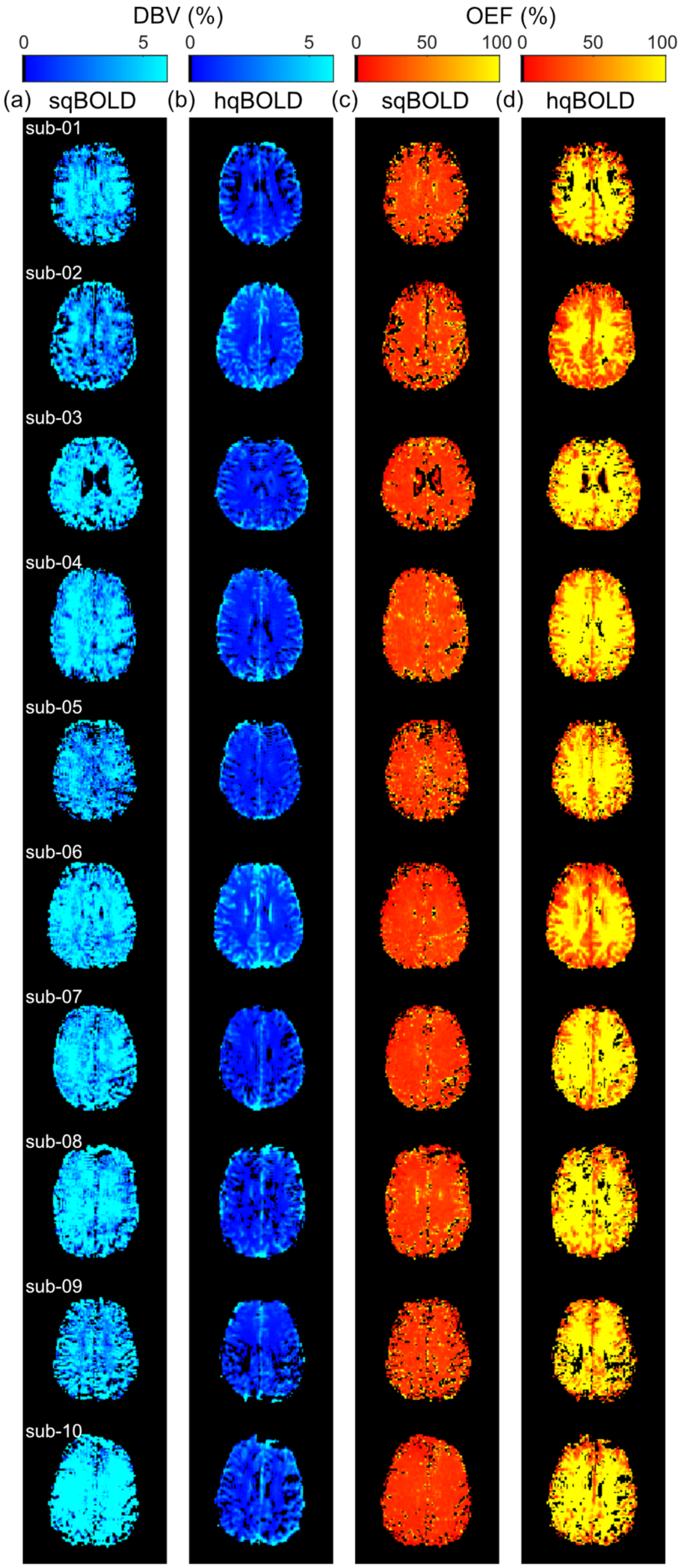

FIGURE S1 A single slice of the streamlined-qBOLD (sqBOLD) and hyperoxia-qBOLD (hqBOLD) data of each of the participants. (a-b) deoxygenated blood volume (DBV) and (c-d) oxygen extraction fraction (OEF) maps.

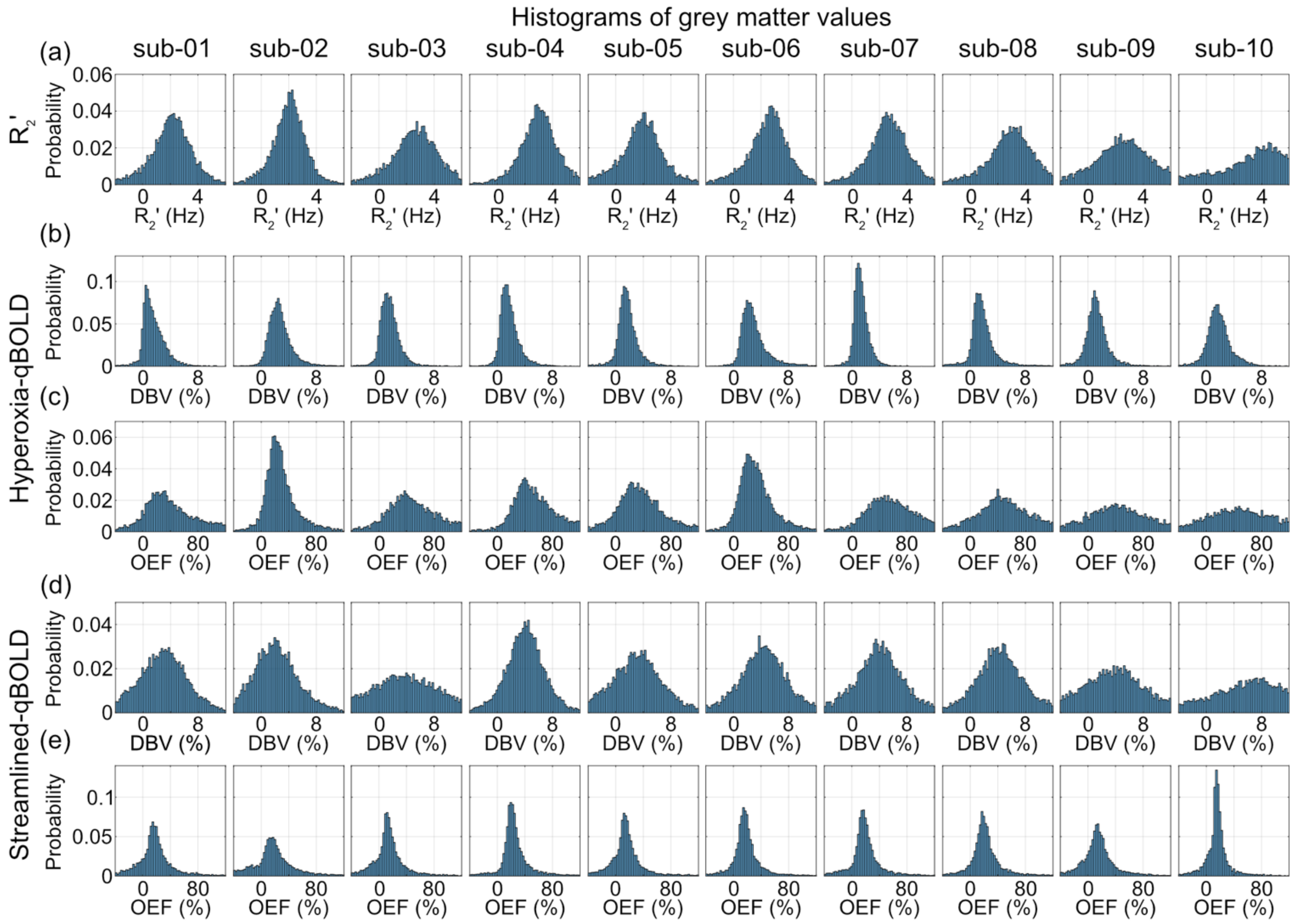

FIGURE S2 Histograms of experimental qBOLD measurements in grey matter for all subjects. (a) the distribution of R_2' values, (b-c) the distribution of deoxygenated blood volume (DBV) and oxygen extraction fraction (OEF) values for hyperoxia-qBOLD (hqBOLD) and (d-e) the distribution of DBV and OEF values for streamlined-qBOLD (sqBOLD).

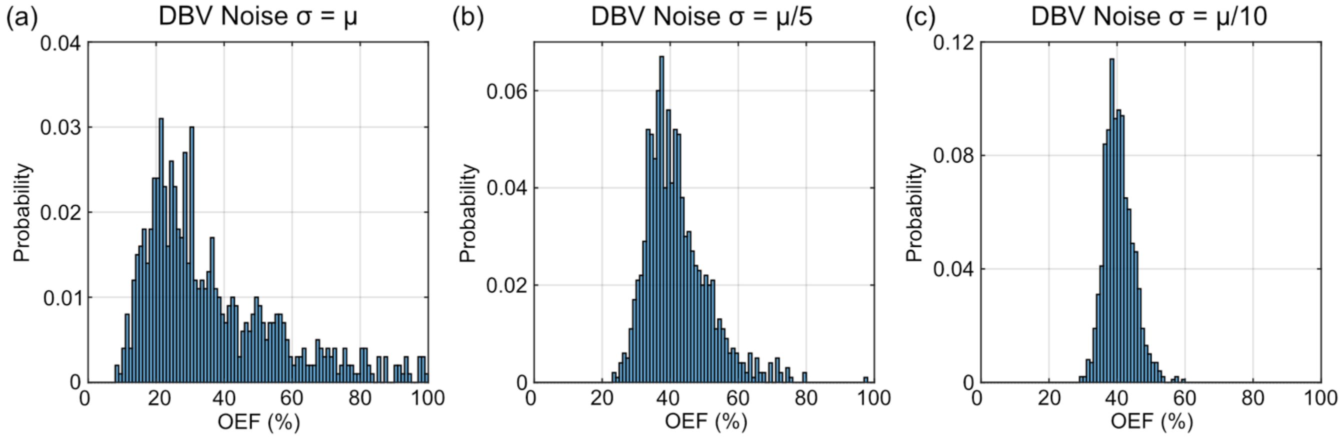

FIGURE S3 Demonstration of the effect of increasing variance in the measurement of DBV on the estimate of OEF. This figure was created by assuming OEF = 40% and DBV = 2%. Gaussian random noise with standard deviation σ was added to the DBV value as a fraction of the mean value μ of DBV. When the noise level is relatively small compared to the mean value of DBV (c), the estimated OEF values are clustered around the simulated OEF of 40%. As the noise level increases (b), the distribution of OEF values becomes broader and skewed and the median value shifts to a lower value than the true value. When the noise level is very high (a), the median value is highly shifted towards zero. Hence the higher noise level present in the sqBOLD estimate of DBV causes a greater level of underestimation than the lower noise hqBOLD measurements.
